# Expression of RECK and matrix metalloproteinase-2 in ameloblastoma

**DOI:** 10.1186/1471-2407-9-427

**Published:** 2009-12-08

**Authors:** Bin Zhang, Jin Zhang, Zhi-Ying Xu, Hong-Liang Xie

**Affiliations:** 1 Department of Oral and Maxillofacial Surgery, the Second Affiliated Hospital, Sun Yat-Sen University, 107 Yanjiang Road West, Guangzhou, Guangdong, 510120, PR China; 2 Department of Internal Medicine, the Second Affiliated Hospital, Sun Yat-Sen University, 107 Yanjiang Road West, Guangzhou, Guangdong, 510120, PR China

## Abstract

**Background:**

Ameloblastoma is a frequent odontogenic benign tumor characterized by local invasiveness, high risk of recurrence and occasional metastasis and malignant transformation. Matrix metalloproteinase-2 (MMP-2) promotes tumor invasion and progression by destroying the extracellular matrix (ECM) and basement membrane. For this proteolytic activity, the endogenous inhibitor is reversion-inducing cysteine rich protein with Kazal motifs (RECK). The aim of this study was to characterize the relationship between RECK and MMP-2 expression and the clinical manifestation of ameloblastoma.

**Methods:**

Immunohistochemistry and reverse transcription-polymerase chain reaction (RT-PCR) were employed to detect the protein and mRNA expression of RECK and MMP-2 in keratocystic odontogenic tumor (KCOT), ameloblastoma and ameloblastic carcinoma.

**Results:**

RECK protein expression was significantly reduced in KCOT (87.5%), ameloblastoma (56.5%) and ameloblastic carcinoma (0%) (P < 0.01), and was significantly lower in recurrent ameloblastoma compared with primary ameloblastoma (P < 0.01), but did not differ by histological type of ameloblastoma. MMP-2 protein expression was significantly higher in ameloblastoma and ameloblastic carcinoma compared with KCOT (P < 0.01). RECK mRNA expression was significantly lower in ameloblastoma than in KCOT (P < 0.01), lower in recurrent ameloblastoma than in primary ameloblastoma, and was negative in ameloblastic carcinoma. MMP-2 mRNA expression was significantly higher in ameloblastoma compared with KCOT (P < 0.01), but was no different in recurrent ameloblastoma versus primary ameloblastoma. RECK protein expression was negatively associated with MMP-2 protein expression in ameloblastoma (r = -0.431, P < 0.01).

**Conclusion:**

Low or no RECK expression and increased MMP-2 expression may be associated with negative clinical findings in ameloblastoma. RECK may participate in the invasion, recurrence and malignant transformation of ameloblastoma by regulating MMP-2 at the post-transcriptional level.

## Background

Odontogenic tumors arise from epithelial, ectomesenchymal or mesenchymal elements that are, or have been, part of the tooth-forming apparatus. Ameloblastoma is a common odontogenic neoplasm estimated to form 36% of all odontogenic tumors in China [1]. Although classified as benign, its locally invasion, distant metastasis and malignant transformation pose a therapeutic challenge in oral and maxillofacial surgery [2]. A better understanding of the molecular mechanisms of ameloblastoma progression may inform the search for adjuvant therapies.

Matrix metalloproteinases (MMPs) play an important role in ameloblastoma invasion, since they are involved in breakdown of the extracellular matrix (ECM) [3]. Among MMPs, MMP-2 has been closely associated with ameloblastoma invasion [4,5]. Reversion-inducing cysteine rich protein with Kazal motifs (RECK) was initially discovered due to its ability to induce reversion in ras-activated fibroblasts. The key action of RECK is to inhibit MMPs, especially MMP-2 and MMP-9 [6]. For this reason, it is important in embryogenesis and other physiological processes. Furthermore, it significantly limits invasion of tumors and inhibits MMP-2. For instance, RECK expression in pancreatic cancer tissue was significantly lower than in adjacent normal tissues and was negatively associated with MMP-2 activation and tumor invasive ability [7]. Restoring RECK expression in human fibrosarcoma HT1080 cells reduced both their invasive ability and the amount of active MMP-2 [8]. To date, no relationship between MMP-2 and RECK in benign ameloblastoma has been documented. Therefore, this study investigated the combinatorial role and association of RECK and MMP-2 in ameloblastoma.

## Methods

### Patients and Tumor Samples

Formalin-fixed paraffin-embedded specimens (dating from 1999 to 2008) were retrieved from the Pathology Service of the Second Affiliated Hospital of Sun Yat-Sen University. Studied were specimens from 69 cases of ameloblastoma (45 males and 24 females; mean age 31.6 years, range 9-72 years; primary cases 45, recurrent cases 24), 6 cases of ameloblastic carcinoma and 16 cases of keratocystic odontogenic tumor (KCOT). The 69 ameloblastoma cases included 22 plexiform ameloblastomas, 29 follicular ameloblastomas, seven unicystic ameloblastomas, four acanthomatous ameloblastomas, three granular cell ameloblastomas and four desmoplastic ameloblastomas. The paraffin embedded tissue samples were cut and mounted on glass slides for immunohistochemical analysis.

In addition, fresh specimens, surgically removed from patients with epithelial odontogenic tumors at the Department of Oral and Maxillofacial Surgery of the Second Affiliated Hospital of Sun Yat-Sen University from March 2007 to December 2008, were collected. The fresh specimens included 22 cases of ameloblastoma (14 males and 8 females; mean age 34.9 years, range 13-72 years; primary cases 12, recurrent cases 10), 2 cases of ameloblastic carcinoma and 16 cases of KCOT. The 22 ameloblastoma cases included eight plexiform ameloblastomas, ten follicular ameloblastomas, two unicystic ameloblastomas, one granular cell ameloblastoma and one desmoplastic ameloblastoma. All fresh tissues were immediately stored in liquid nitrogen for further analysis. Among these cases, ameloblastic carcinomas and KCOT were used as controls. Diagnosis and classification of all specimens was confirmed by histopathologic examination according to the WHO classification. All patients gave written informed consent, and the approval of the Sun Yet-sen University Ethics Committee was obtained.

### Immunohistochemical staining

Immunohistochemical staining was performed using the 2-step plus poly-HRP method as described previously [9]. Briefly, one representative section of the tissue was cut at 4 μm and placed on poly-L-lysine coated slides. The slides were deparaffinized, rehydrated, immersed in 10 mM sodium citrate buffer (pH 6.0) and pretreated in a microwave oven for 10 min, followed by a 10-minute rinse with phosphate-buffered saline (PBS). After blocking with 3% hydrogen peroxide for 10 min at room temperature, the slides were incubated at 4°C overnight with primary antibodies: anti-RECK (1:50 goat monoclonal antibody, Santa Cruz Biotechnology, Inc. Santa Cruz, CA) and anti-MMP-2 (1:200 mouse monoclonal antibody, Santa Cruz Biotechnology, Inc. Santa Cruz, CA). Afterwards, the slides were stained with the 2-step plus Poly-HRP Anti-Goat IgG Detection System (ZSGB-Bio, Beijing, China) for RECK or 2-step plus Poly-HRP Anti-Mouse IgG Detection System (ZSGB-Bio, Beijing, China) for MMP-2. After visualization of the reaction with the DAB chromogen, the slides were counterstained with haematoxylin and covered with a glycerin gel. As positive controls, a colon cancer specimen was used for RECK and a breast cancer specimen for MMP-2 (patient consent obtained). Negative controls consisted of tissue sections incubated with PBS instead of the primary antibody.

### Evaluation of immunohistochemical staining

Sections were evaluated by two blinded, experienced investigators who provided a consensus opinion of stain patterns by light microscopy. Immunohistochemical staining of cells was assessed according to stain intensity and proportion of cells stained. Stain intensity was graded using a 4-point scale: 0, no staining; 1, mild staining; 2 moderate staining; and 3, intense staining. The proportion of cells stained was assessed using a semiquantitative 4-point scale: 0, no cell staining in any microscopic fields; 1, <25% staining; 2, 25-50% staining; and 3, >50% staining. The combined score (extension plus intensity) was assessed as follows: <2, negative staining or low staining (-); 2 and 3, moderate staining (+); and ≥4, strong staining (++). A combined score equaling or exceeding (+) was defined as positive for RECK and MMP-2.

### Reverse transcription-polymerase chain reaction (RT-PCR) and image analysis

Gene expression studies were conducted using gene-specific primers for RECK and MMP-2. Total RNA was extracted from the frozen tissues and subjected to reverse transcription-polymerase chain reaction (RT-PCR) as follows. Total RNA from 100 mg tissue samples was obtained by the Trizol method (GIBCO-BRL, Grand Island, NY) according to the manufacturer's instructions, and an Access RT-PCR system (Promega, Madison, WI) was used to amplify the products. RT-PCR of a single target RNA was performed in a single tube with Avian Myeloblastosis Virus reverse transcriptase (AMV RT) for first-strand DNA synthesis; Thermus flavus (Tfl) DNA polymerase for second-strand complementary DNA (cDNA) synthesis; and 5 M oligodeoxythymidylate, 10 mM deoxynucleoside triphosphate and 1 mM Mg2+ for DNA amplification in a volume of 50 μL. RT-PCR was performed in a DNA Thermal Cycler 480 (Perkin Elmer Corp., Norwalk, CT) at the following cycles: 45 min RT at 48°C, 5 min AMV RT inactivation and RNA/cDNA/primer denaturation at 94°C, 30 sec denaturation at 94°C, 30 sec annealing at either 59°C (β-acting) or 55°C (RECK, MMP-2), and a 100 sec (RECK and MMP-2) or 120 sec (β-acting) extension at 72°C. Thirty (RECK, MMP-2) or 35 (β-acting) cycles were conducted per amplification of each PCR product. The linear range for PCR conditions was tested in pilot experiments and was 30-40 cycles for RECK, MMP-2 and β-acting. All subsequent reactions were carried out within this range.

The PCR products were fractionated by 1.5% agarose gel electrophoresis (agarose-1000, GIBCO-BRL, Grand Island, NY) and stained with 0.5 μg/mL ethidium bromide (GIBCO-BRL, Grand Island, NY), and the identity of the PCR products confirmed using a 100-bp ladder (Promega, Madison, WI) as the DNA standard. Each PCR reaction was replicated a minimum of three times. Primer sequences were: RECK (NM021111): sense-primer 5'-TAACCAAATGTGCCGTGATG-3', antisense 5'-TCCAAGGCAATAGCCAGTTC-3'; MMP-2 (NM004530): sense-primer 5'-GATGCCGCCTTTAACTGG-3', antisense 5'-TCAGCAGCCTAGCCAGTCG-3'; and β-acting (NM001101): sense-primer 5'-GATGAGATTGGCATGGCTTT-3', antisense 5'-CTCAAGTTGGGGGACAAAAA-3'. Amplification products of the RNAs coding for RECK, MMP-2 and β-acting were 207, 279, and 431 bp, respectively. Quantitation was performed by densitometry, with β-acting used as an internal control for RT-PCR reactions and the products analyzed on a 1.5% agarose minigel system. A computerized image analysis system (Kontron IBAS2.0, Munich, Germany) was used to quantify band intensity. Results were evaluated as a relative unit determined by normalization of the optical density (OD) of RECK or MMP-2 band to that of β-acting band (the ratio of RECK/β-acting and MMP-2/β-acting, respectively).

### Statistical analysis

Results of RECK and MMP-2 protein expression are described with positive ratio, with differences between groups tested for significance using Fisher's exact and Pearson Chi-square tests. RECK and MMP-2 mRNA expression are expressed as mean ± standard deviation (SD), and the Wilcoxon rank sum test and T-test were used to analyze differences in results between groups. The correlation in protein or mRNA expression of RECK and MMP-2 was analyzed using Spearman's rank correlation. Statistical analysis was performed using SPSS version 15.0 (SPSS Inc., Chicago, IL). P values < 0.05 were considered significant.

## Results

### Expression of RECK and MMP-2 protein in KOCT, ameloblastoma and ameloblastic carcinoma

**Figure 1:**
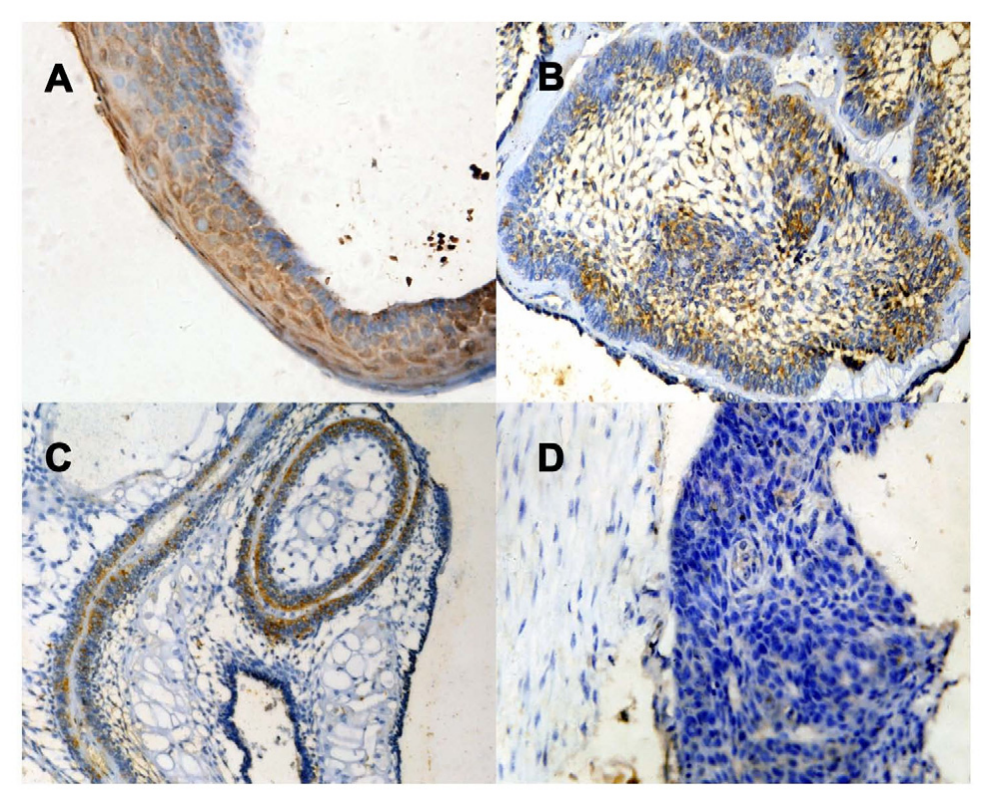
**A representative immunohistochemical reactivity for RECK**. (A) Keratocystic odontogenic tumor showing strong reactivity (× 400); (B) Follicular ameloblastoma showing strong reactivity in central polyhedral cells and weak in peripheral columnar cells (× 200); (C) Plexiform ameloblastoma showing reactivity in peripheral columnar cells and nearly no expression in central polyhedral cells (× 200); (D) Ameloblastic carcinoma showing no expression in tumor cells (× 400).

Immunoreactivity for RECK was detected in nearly all epithelial cells of KOCT, in central polyhedral cells of follicular ameloblastoma and in peripheral columnar cells of plexiform ameloblastoma, but not in ameloblastic carcinoma cells (Figure 1). RECK expression gradually significantly decreased in KCOT (87.5%), ameloblastoma (56.52%) and ameloblastic carcinoma (0%, *P *< 0.01), and was significantly higher in primary ameloblastoma (71.11%) than in recurrent ameloblastoma (29.17%, *P *< 0.01), but no significant difference was seen between histological types (Table 1).

**Table 1:** Immunohistochemical reactivity for RECK and MMP-2 in KCOT, AB and AC

	n	RECK				MMP-2			
			
		-	+	++	*P*	-	+	++	*P*
KCOT	16	2	5	9	0.000	13	0	3	0.000
AB	69	30	22	17		11	23	35	
AC	6	6	0			0	1	5	
									
Primary AB	45	13	17	15	0.001	8	19	18	0.82
Recurrent AB	24	17	5	2		3	4	17	

KCOT; keratocystic odontogenic tumor, AB; ameloblastoma, AC; ameloblastic carcinoma.

**Figure 2:**
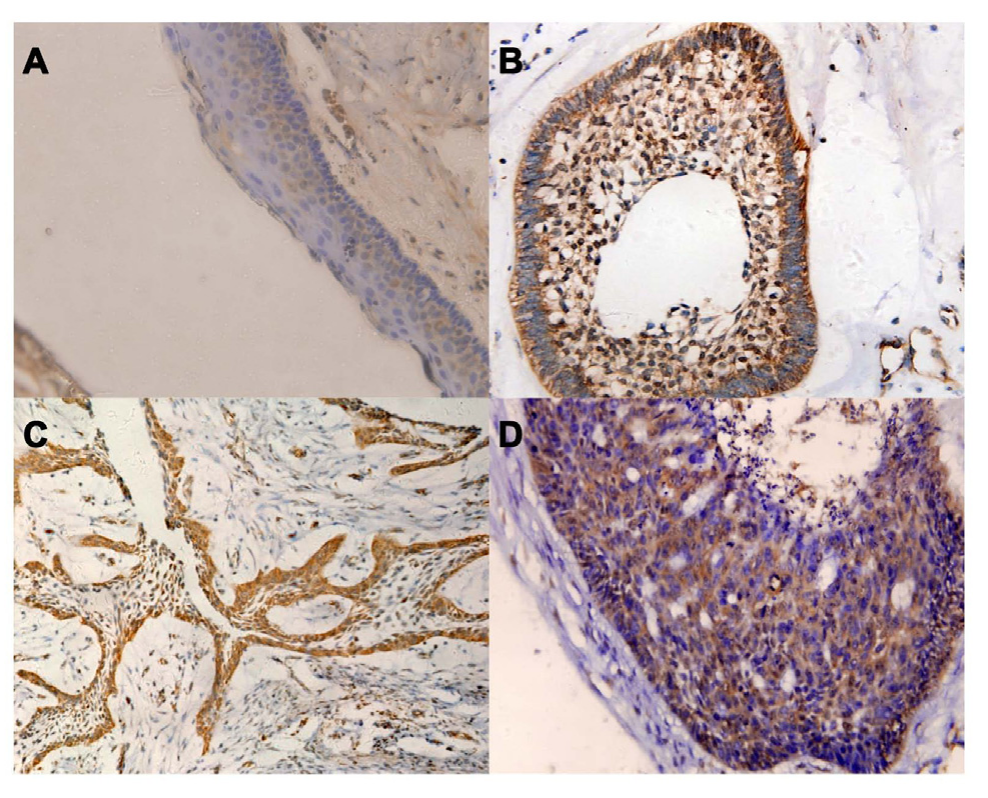
**A representative immunohistochemical reactivity for MMP-2**. (A) Keratocystic odontogenic tumor showing moderate reactivity in prickle cells (× 400); (B) Follicular ameloblastoma showing strong reactivity in peripheral columnar cells (× 400); (C) Plexiform ameloblastoma showing strong reactivity in peripheral columnar cells (× 200); (D) Ameloblastic carcinoma showing strong reactivity in tumor cells (× 400).

Immunoreactivity for MMP-2 was mainly detected in prickle cells of KCOT, peripheral columnar or cuboidal cells of ameloblastoma and ameloblastic carcinoma cells (Figure 2). MMP-2 expression was significantly higher in ameloblastic carcinoma (100%) and ameloblastoma (84.06%) than in KCOT (18.75%, *P *< 0.01), but was not significantly different in primary ameloblastoma (82.22%) versus recurrent ameloblastoma (87.5%) or between histological types (Table 1).

### Expression of RECK and MMP-2 mRNA in KOCT, ameloblastoma and ameloblastic carcinoma

**Figure 3:**
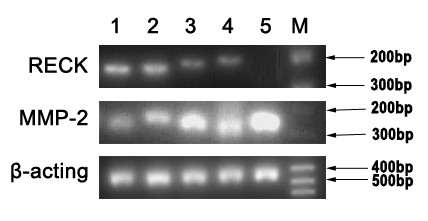
**Representative gel pictures showing the mRNA expression of RECK and MMP-2**. M, DNA marker; 1 and 2, Keratocystic odontogenic tumor; 3 and 4, Ameloblastoma; 5, Ameloblastic carcinoma.

RECK mRNA was detected in all KCOT and ameloblastoma samples, but not in either ameloblastic carcinoma sample. MMP-2 mRNA was detected in all KCOT, ameloblastoma and ameloblastic carcinoma samples, and strongly expressed in both ameloblastic carcinoma samples (Figure 3). Because of the lack of ameloblastic carcinoma cases, their data were not analyzed statistically. Relative expression levels of RECK mRNA were significantly lower in ameloblastoma than in KCOT (*P *< 0.01), but significantly higher for MMP-2 mRNA (*P *< 0.01). In ameloblastoma, relative expression levels of RECK mRNA were significantly lower in recurrent ameloblastoma than in primary ameloblastoma (*P *< 0.05), but levels of MMP-2 mRNA did not differ to a statistically significant extent between recurrent ameloblastoma and primary ameloblastoma. Relative expression levels of RECK and MMP-2 mRNA did not differ statistically between follicular ameloblastoma and plexiform ameloblastoma (Table 2).

**Table 2:** Relative expression levels of RECK and MMP-2 mRNA in KCOT and AB

	n	RECK/β-acting		MMP-2/β-acting	
			
		mean ± SD	*P*	mean ± SD	*P*
KOCT	16	1.361 ± 0.145	0.000	0.099 ± 0.047	0.000
AB	22	0.131 ± 0.023		1.425 ± 0.174	
					
Primary AB	12	0.165 ± 0.033	0.016	1.577 ± 0.249	0.959
Recurrent AB	10	0.091 ± 0.028		1.561 ± 0.208	

KCOT; keratocystic odontogenic tumor, AB; ameloblastoma.

### The relationship between RECK and MMP-2 in ameloblastoma

In ameloblastoma, protein expression in RECK and MMP-2 were inversely proportional (r = -0.431, *P *< 0.01, Table 3), but mRNA expression of RECK and MMP-2 were not correlated (r = 0.367, *P *> 0.05).

**Table 3:** Correlation of RECK and MMP-2 protein expression in ameloblastoma

	RECK				
			
MMP-2	-	+	++	*r*	*P*
-	1	2	8	-0.431	0.000
+	10	6	7		
++	19	14	2		

## Discussion

Many studies have verified that MMP-2 plays an important role in tumor invasion [10,11]. Our and other studies also showed that the high expression and activity of MMP-2 is related to a more aggressive behavior in ameloblastoma [4,5,12]. RECK can inhibit MMP-2 expression and activity as an endogenous inhibitor of MMP-2 in physiological and pathological processes. Previous studies have shown that high RECK expression can inhibit the invasion potential of some tumors by inhibiting MMP-2 [7,8]. However, few reports have delineated the combinatorial role and association of RECK and MMP-2 in ameloblastoma.

RECK is an anti-oncogene inversely related to tumor invasion that is downregulated in many tumors. Takeuchi and colleagues [13] found RECK downregulated in most colon cancer specimens compared with surrounding normal tissue, and low RECK expression in lymph nodes with a high metastatic rate. Mori's study showed significant inhibition of the invasive potential of HT1080 cells and their mutant Rzmet-2 after being transfected with RECK plasmid [14]. In our study, RECK protein and mRNA expression were gradually reduced in KCOT, primary ameloblastoma, recurrent ameloblastoma and ameloblastic carcinoma, similar to the results by Kumamoto et al. [15], who also found RECK protein expression downregulated in ameloblastoma compared with tooth germ. Our results indicated that the protein expression and the transcriptional levels of RECK were sequentially lower or absent as odontogenic tumor cells increased in aggressiveness, indicating that RECK expression levels may be correlated with clinical outcomes in ameloblastoma.

RECK may inhibit tumor invasion and metastasis by inhibiting the expression and activity of MMP-2. MMP-2 is one of the most important proteolytic enzymes that degrade basement membrane and extracellular matrix during tumor invasion and metastasis [16]. Increased expression and activity of MMP-2 could promote tumor invasion ability [17-19]. Similar findings have been described in our and other studies of ameloblastoma, indicating that high MMP-2 expression and activity is related to a more aggressive infiltrative behavior in ameloblastoma [4,5,12]. Our study showed that MMP-2 protein expression and transcriptional levels sequentially increased in KCOT, ameloblastoma and ameloblastic carcinoma, and expression levels of MMP-2 protein and mRNA were significantly higher in ameloblastoma than in KOCT, or just the opposite of RECK, indicating that MMP-2 protein expression and transcription sequentially increased with the aggressiveness of odontogenic tumor cells. These results demonstrate that increased MMP-2 expression may be implicated in poor clinical prognosis and may be relation to lower or even no expression of RECK in ameloblastoma.

RECK is thought to inhibit MMP-2 at the post-transcriptional level. Masui et al. [7] showed that the expression of RECK was inverse correlation to MMP-2 activity and the invasive capability of pancreatic cancer. Yoshida et al. [20], using RECK mRNA-targeting siRNA interference, reported that the expression of MMP-2 protein and its consequent invasive capability increased in pituitary adenomas cells after RECK had been downregulated. Mori found [14] that MMP-2 activity, but not its mRNA expression, was significantly downregulated in HT1080 cells after they were transferred into the RECK plasmid. Similarly, our results showed a negative correlation between RECK and MMP-2 protein expression, but no correlation between RECK and MMP-2 mRNA expression in ameloblastoma, indicating that RECK may reduce aggression of ameloblastomas by inhibiting MMP-2 expression at the post-transcriptional level.

## Conclusion

In summary, lower or no expression of RECK and increased expression of MMP-2 may be associated with worse clinical outcomes in ameloblastoma, and RECK may help modify the behavior of ameloblastomas by regulating MMP-2 at the post-transcriptional level. Further research is warranted to elucidate the further mechanisms involved so as to develop appropriate therapeutic interventions.

## List of abbreviations used

MMP: Matrix metalloproteinase; ECM: extracellular matrix; RECK: reversion-inducing cysteine rich protein with Kazal motifs; RT-PCR: reverse transcription-polymerase chain reaction; KCOT: keratocystic odontogenic tumor; AB: ameloblastoma; AC: ameloblastic carcinoma; PBS: phosphate-buffered saline; AMV: Avian Myeloblastosis Virus; Tfl: Thermus flavus; cDNA: complementary DNA; mRNA: message RNA; OD: optical density.

## Competing interests

The authors declare that they have no competing interests.

## Authors' contributions

BZ, JZ, ZYX and HLX were responsible for the experimental design and completion of all laboratory work represented in this manuscript. The manuscript was drafted by BZ and JZ. All authors have read and approved the final manuscript.

## Pre-publication history

The pre-publication history for this paper can be accessed here:

http://www.biomedcentral.com/1471-2407/9/427/prepub
